# New Oral Formulation and in Vitro Evaluation of Docetaxel-Loaded Nanomicelles

**DOI:** 10.3390/molecules21091265

**Published:** 2016-09-21

**Authors:** Atefeh Hekmat, Hossein Attar, Ali Akbar Seyf Kordi, Maryam Iman, Mahmoud Reza Jaafari

**Affiliations:** 1 Department of Chemical Engineering, Science and Research Branch, Islamic Azad University, Tehran 1477893855, Iran; atefehhekmat@yahoo.com (A.H.); attar.h@srbiau.ac.ir (H.A.); a-seifkordi@srbiau.ac.ir (A.A.S.K.); 2 Chemical Injuries Research Center, Baqiyatallah University of Medical Sciences, Tehran 193955487, Iran; iman1359@yahoo.com; 3 Nanotechnology Research Center, School of Pharmacy, Mashhad University of Medical Sciences, Mashhad 917751365, Iran

**Keywords:** nanomicelle, docetaxel, Taxotere^®^, encapsulation, stability, C-26 cell line, in vivo, in vitro

## Abstract

Intravenous administration of Taxotere^®^ (a commercial form of docetaxel, DTX) leads to many problems such as hypersensitivity, hemolysis, cutaneous allergy, and patient refusal due to its prolonged injection. The oral absorption of DTX is very low due to its hydrophobic nature. The purpose of this study was to prepare and carry out an in vitro evaluation of DTX-loaded nanomicelles for oral administration in order to increase the oral delivery of DTX. Studied formulations were prepared with the two surfactants Tween 20 and Tween 80 and were characterized for their particle size, zeta potential, stability, encapsulation efficiency, stability studies in gastric fluid and intestinal fluid, toxicity studies in C26 colon carcinoma cell line, and cellular uptake. The prepared nanomicelles with particle size of around 14 nm and encapsulation efficiency of 99% were stable in gastric fluid and intestinal fluid for at least 6 h and IC50 decreased significantly after 72 h exposure compared to that of Taxotere^®^. Nanomicelles increased the water solubility of DTX more than 1500 times (10 mg/mL in nanomicelles compared to 6 µg/mL in water). Results of this study reveal that the new formulation of DTX could be used for the oral delivery of DTX and merits further investigation.

## 1. Introduction

Docetaxel (DTX) is an antineoplastic compound from the anthranilates group [1]. It can be prepared through the semi-synthesis of taxol which is extracted from the European yew tree [2,3,4,5,6,7,8,9]. DTX has a wide range of antitumor ability. It is used as a treatment for acute leukemia, Hodgkin and non-Hodgkin lymphoma, colorectal, breast, stomach, lung, and prostate cancers, as well as solid tumors [2,3,4,5,6,7,8,9,10,11].

The pharmacological mechanism of DTX with microtubule cytoskeleton stability involves inhibition of microtubule depolymerization at the G2/m cell cycle stage [2,10,11]. The tumor- inhibitory efficacy of DTX is almost two times greater than that of common cancer drugs such as paclitaxel [2,12,13,14]. Low solubility is one of the limitations of using docetaxel in treatment. The low hydrosolubility and high toxicity of this drug result in decreasing bioavailability and decreasing efficacy [2,15,16].

Taxotere^®^ is the commercial version of this drug. Taxotere^®^ is administrated intravenously to the patients as an 13% ethanol solution during 4 h. The intravenous administration leads to many problems such as cutaneous allergy, and patient refusal due to its prolonged injection [2,4,10,17,18,19]. Many studies have been conducted on producing oral formulations of DTX to prevent the abovementioned issues, patient discomfort, inability to change administration schedule, need for abundant nursing staff, and high treatment costs [20,21,22].

In order to improve the therapeutic index and to reduce the adverse effects of DTX, new generation drug delivery systems such as liposomes, solid lipid nanoparticles (SLNs), and micelles [2,3,4,12] have been used. In addition, the oral formulation of new carriers has advantages over its commercial forms [10]. The low bioavailability of DTX can be reduced by using stabilizers and surfactants to develop adequate formulations. Increasing the bioavailability is the most important point in the oral formulation of DTX. Many scientists have reported that the poor bioavailability of DTX was due to the membrane transporter P-glycoprotein (ABCB1), as well as low permeability and solubility [23,24]. P-glycoprotein inhibitors, drug dissolution in the self-emulsifying targeting systems, and nanocarriers such as micelles have been used in order to solve the poor bioavailability issues [2,25].

The gastrointestinal tract (GI) is lined with a layer of mucus, which provides a barrier to acid in the stomach [26,27]. A space (unstirred water layer) exists between the mucus itself and the apical surface of the epithelial cell monolayer of GI. It should be realized that a drug must first diffuse across the mucus layer before the absorption across the epithelia is possible [26,28]. The aqueous boundary or unstirred water layer can be rate limiting for highly lipophilic compounds [26]. Lipophilic compounds with very low water solubility like DTX do not dissolve in the unstirred water layer and that is why their bioavailability is low after oral administration [2,3,4,5,6,7,8,9,10,11]. However, nanomicelles can increase the water solubility of DTX and bypass the unstirred water layer and increase the oral absorption of DTX.

Nanomicelles are produced from ionic and nonionic surfactants. Surfactants are surface active agents which are produced from a hydrophilic polar compartment as the head part and a hydrophobic non polar compartment as the tail part [2,29,30]. Nanomicelles are produced by compaction of non-covalent single monomers of surfactants, which contain hydrophobic compartments and amphiphilic shells that deliver drug to the patients’ cell membrane [2,10,31,32]. In addition, the solubility of poorly soluble drugs in water can be achieved [2,4,7,8,9,10,11,14]. DTX encapsulation in such a system might lead to higher stability and more proper tissue distribution [2,3,4,5,6,7,8,9,10,11,12,13,14,15,16].

Core-shell micelles have a hydrophobic core and hydrophilic shell. The drug is loaded in the core with high solubility whereas the shell or corona has hydrophilic characteristics which inhibit drug inactivation in the gastrointestinal media.

Micelles have high solubility capacity (drug loading) for low hydrosolubility drugs, and as a direct carrier of drug in comparison with surfactant (with low molecular weight) they have significantly lower critical concentrations, and longer life times (higher thermodynamic stability) [2,10]. Surfactant (Tween 80) was directly used to increase drug solubility in the commercial form of DTX. Based on these advantages, micelles have been used as cancer drug carriers for oral administration [2,10].

Tween 80 surfactant has usually been used in formulations because of its high ability to solubilize DTX and perfect release behavior, and in combination with a suitable stabilizer, it can inhibit P-gp-mediated multidrug resistance and increase the bioavailability of DTX [33].

The present research aimed at the preparation and evaluation of two MCT oil DTX-loaded nanomicelles prepared with the two surfactants Tween 20 and Tween 80. Two nanomicelles were prepared with Tween 80 and Tween 20 without DTX and characterized for particle size, zeta potential, stability, encapsulation efficiency, stability studies in gastric fluid and intestinal fluid, toxicity studies in C26 colon carcinoma cell line, and cellular uptake.

## 2. Results

### 2.1. Nanomicelles Evaluation

Table 1 shows the content of the prepared formulations.

**Figure 1:**
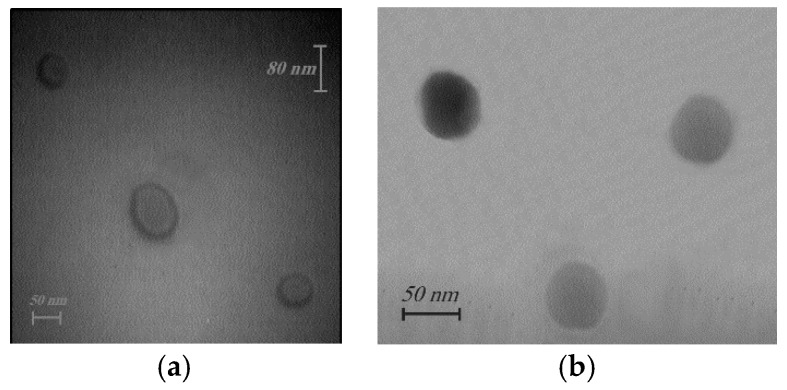
TEM pictures of prepared DTX-loaded nanomicelles of formulation I (**a**) and formulation III (**b**).

TEM pictures demonstrated the spherical shape of the prepared nanomicelles (Figure 1).

The mean particle size of the Tween 80 nanomicelles was 14.03 ± 1.23 nm, and that of the Tween 20 nanomicelles was 132.55 ± 12.88 nm. The zeta potential of Formulation I was −9.45 mV and the zeta potential of Formulation III was −6.09 mV (Table 2). The Z-average and polydispersity index of the Tween 80 formulations (I and II) was less than that of Tween 20 formulations (III and IV) which showed that the size and homogeneity of the Tween 80 formulation were better.

### 2.2. Determination of Encapsulation Efficacy

Encapsulation efficacy of Formulation I was calculated 99.01% by HPLC method. Results showed that almost all the DTX was encapsulated in the nanomicelle and almost there were no free drug in the formulation medium.

### 2.3. Stability Studies

**Figure 2:**
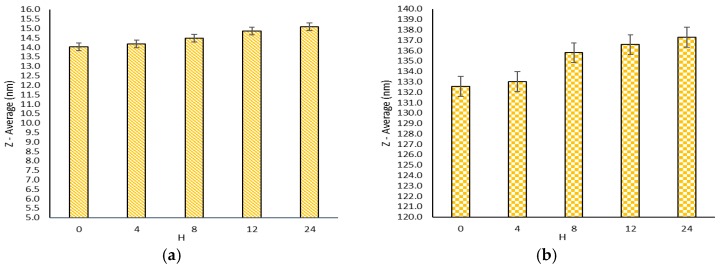
Particle size changes in formulation I (**a**) and formulation III (**b**) at room temperature (condition A).

Stability study results of the prepared formulations showed that there were no significant differences in particle size and zeta potential in all three conditions: (A) at room temperature (short term stability); (B) at 2–8 °C (long term stability); and (C) in SGF/SIF. Figure 2 shows the particle size changes in Formulation I (Figure 2a) and Formulation III (Figure 2b) at room temperature (under condition (A). The results show that Formulation I was completely stable, however, Formulation III became bigger but the differences were not significant. The zeta potential for Formulation I was −10 mV and it was −6.53 mV for Formulation III.

**Figure 3:**
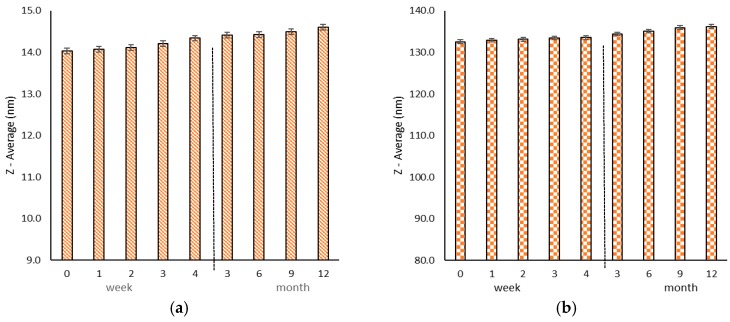
Particle size changes in formulation I (**a**) and formulation III (**b**) at 2–8 °C (condition B).

Figure 3 shows particle size changes in Formulation I (Figure 3a) and Formulation III (Figure 3b) at 2–8 °C (condition (B). The zeta potentials of the two formulations were almost the same as their previous values (under real storage conditions: Formulation I, −9.92 mV, and Formulation III, −6.29 mV) with no significant changes during the storage. The results of Figure 3 demonstrate that the formulations were stable for more than 12 months when stored in a refrigerator. Table 3 shows the long term encapsulation efficacy of the DTX in the nanomicelles after 12 months and the short term efficacy after a 24 h stability study under conditions (A) and (B).

**Figure 4:**
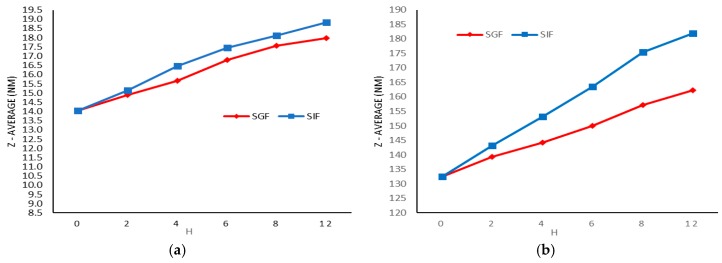
Particle size changes in formulation I (**a**) and formulation III (**b**) in SGF&SIF (condition C).

Figure 4 demonstrates particle size changes in Formulation I (Figure 4a) and Formulation III (Figure 4b) in SGF & SIF (condition C). In SGF the nanomicelles showed no significant changes in size after 4 h, and reached 18 nm after 8 h. Since the micelles remained stable for 4 h in SGF and SIF, the nanomicelles were suitable for the oral delivery of the DTX.

### 2.4. Cellular Toxicity Studies

**Figure 5:**
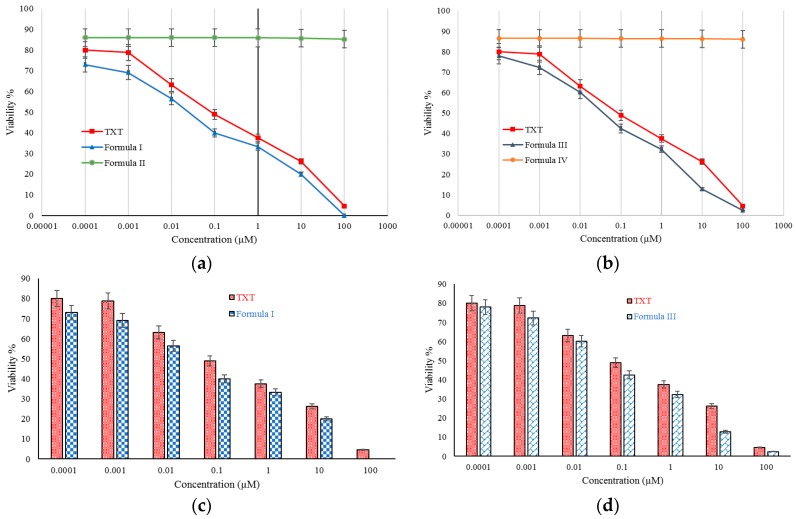
Cytotoxicity of TXT and formulation I to IV on C26 cell lines after 72 h exposure to 0.0001~100 M samples by MTT assay. (**a**) viability of C26 cell lines for formulation I, II, and TXT; (**b**) viability of C26 cell lines for formulation III, IV, and TXT; (**c**) viability percentages of C26 cell lines for formulation I and TXT; (**d**) viability percentages of C26 cell lines for formulation III and TXT.

**Figure 6:**
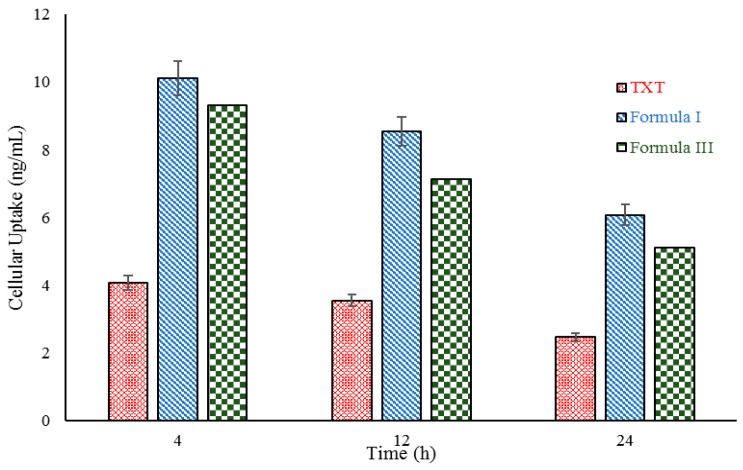
C26 cell DTX uptake in exposure of formulations I, III, and 100 mg/mL TXT at 4, 12, and 24 h.

Figure 5a,b shows the viability of C26 cell lines by MTT test. The formulations had concentrations of 0.0001 µM to 100 µM, and the analysis was performed after 72 h. As is seen in Figure 6, Formulation II (empty nanomicelles) had little effect on the viability of the C26 cells; however, almost 100% of C26 cells died on exposure of 100 µM concentration of Formulation I, and 4.5% of C26 cells survived exposure to the commercial form TXT at the same concentration. This difference was not significant (*p* > 0.05). In all other concentrations, formulation I resulted in a significantly higher death rate than TXT at the same concentration.

Figure 5b, like Figure 5a, demonstrates that Formulation IV didn’t have considerable toxicity, and more than 86% of C26 cells remained alive. In addition, 2.37% and 4.5% of C26 cells survived exposure to 100 µM of Formulation III and TXT, respectively. At all other concentrations, Formulation III resulted in a higher C26 cell death rate. In summary, Figure 5c,d shows that formulations I and III resulted in higher percentages of C26 cell death than TXT.

### 2.5. IC_50_

Table 4 displays the IC_50_ values of formulations I and III in comparison with TXT. The IC_50_ for Formulation I was lower than for TXT and Formulation III.

### 2.6. In Vitro Cell Uptake

DTX accumulation in C26 cells has been measured for the evaluation of cell uptake efficiency through a HPLC method, and it was compared with TXT uptake by the cells. The results showed that C26 cells are more capable of accumulating Formulation I than TXT. As seen in Figure 6, C26 cell uptake of formulations I and III were more than for TXT at all periods of 4, 12, and 24 h, and the uptake differences were significant.

## 3. Discussion

DTX is an anti-cancer drug for the treatment of breast, stomach, lung, and prostate cancers which has interested many scientists [34,35,36]. Poor solubility, low bioavailability, and the high toxicity of docetaxel result in many side effects, low efficacy, and limited consumption. The commercial form of this drug (Taxotere^®^) causes hypersensitivity due to hemolysis and may disturb patients because of its prolonged administration time. In this study, two different oral formulations of DTX were prepared and compared with the commercial product TXT. Many studies show that the compatibility of the solvent (drug) in the main structural block is an effective factor in the loading capacity and encapsulation efficacy of nanomicelles [2,10,37]. In this experiment, constant amounts of 55% wt. of Tween was used for both Tween 20 and 80 in the formulations for oral administration.

TEM images clearly demonstrate the hard surface and spherical shape of the prepared nanomicelles [2,3,10,38]. This morphology protects the drug by minimizing the drug’s contact with aqueous media, while creating the longest diffusion pathways [38]. The particle sizes of these nanomicelles are approximately 14 nm. Nanomicelles smaller than or equal to 100 nm stay longer in blood [2,39].

The extent and rate of absorbed drug in the gastrointestinal tract are dictated by the carrier size and surface properties. The specific area increases due to the small size of nanomicelles, and the contact area with the epithelial surface increases. Therefore, it has a higher potential for non-specific absorption into the cells or endocytosis by receptors [40]. This means small particle size increases enterocyte absorption. Studies revealed that nanocarriers smaller than 50–100 nm are absorbed by enterocytes. Docetaxel nanomicelles pass through the lymphoid system, therefore, they inhibit liver metabolic first pass effects such as metabolism by cytochrome P450 3A4 (CYP 3A4). This leads to an increase in the specific concentration of the drug in blood in comparison with injection administration and decreases cancer cell growth [40].

**Figure 7:**
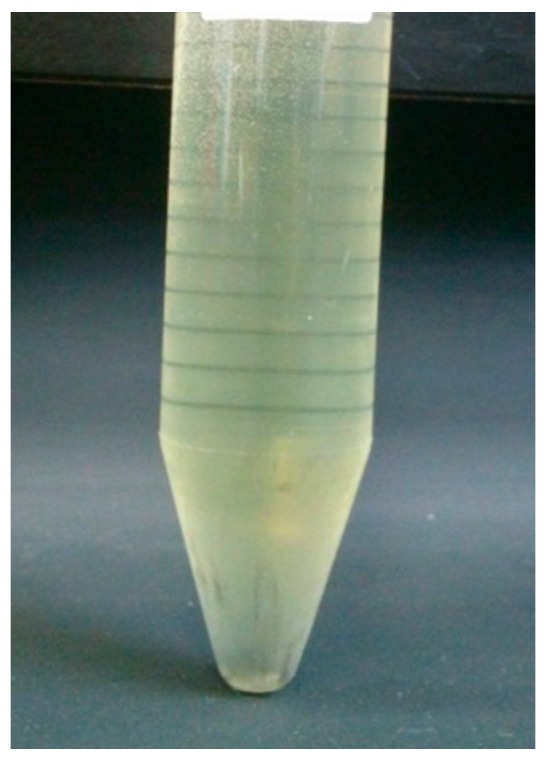
DTX-loaded nanomicelle solution.

The effect of particle size on uptake rate has been assessed in the human digestive system. Table 2 lists the particle sizes of formulations I to IV. The particle size of Tween 80 nanomicelles is smaller than that of Tween 20 ones although Tween 20 and Tween 80 with similar hydrophilic-lipophilic balance (HLB) have similar DTX solubility (Tween 20 = 16.6, Tween 80 = 15) and their related nanomicelles have high transparency due to their highly hydrophilic characteristics (Figure 7) [34]. Therefore, Tween 80 is more suitable for preparing nanomicelles which should be absorbed by digestive system, and the particle size stability can be improved by the negative zeta potential of the formulations [33]. Negative zeta potential with an electrostatic repulsion specification inhibits particle accumulation and leads to better physical stability. Micelles with negative or neutral charges have longer blood half-life [41].

The OH group by itself leads to negative charges on DTX. Therefore, in formulations without DTX (formulations II and IV), the zeta potentials were 5 mV higher than for DTX-loaded formulations (I and III) [42].

Reviewing the encapsulation calculation reveals that Tween 80 nanomicelles are capable of trapping DTX and delivering it to the patients. It was observed that Tween 20 has less effect on DTX encapsulation.

Stability studies after maintaining the formulations at room temperature for 24 h and for 12 months at 2–8 °C show that there were no significant changes in particle sizes, zeta potential, and encapsulation efficacy. Therefore, it can be concluded that the physical stability of nanomicelles is satisfactory. Furthermore, reevaluating EE shows 99% ± 0.05% and 98.9% ± 0.1% of drug contents remained in the micelle after 24 h at room temperature and 12 months in 2–8 °C, and there was no significant change in particle size. Reevaluating EE reveals that the prepared nanomicelles had suitable stability.

Proper drug delivery to the patient’s organs is the most important application of carrier stability [10]. Thus, nanomicelles should have sufficient stability against fast decomposition and dilution in the digestive system. Stability studies of nanomicelles at 37 °C in SGF (pH = 1.6) and SIF (pH = 6.5) have been investigated for 12 h accordingly. Results show that in the first 2 h, the particle size showed minor changes in SGF. However, the nanomicelles’ size in SIF during 6 h increased. As nanomicelles stay less than 6 h in the intestine [10], the physical stability of nanomicelles is satisfactory. In addition, the results of Formulation I demonstrate that using Tween 80 led to a formulation with more stability in the intestinal medium. The results in this study demonstrated that nanoparticles were stable in the gastrointestinal tract and could protect loaded DTX against media pH, enzymatic dissolution, and drug efflux pumps [40].

Cellular toxicity studies of the prepared formulations show that in MTT studies, Formulation I had more effect on C26 cell lines than the commercial product and Formulation III. Due to the probability of micelles connecting to the cell membrane, entering the cytosol, and releasing drug, DTX cellular adsorption from formulations I and III is significantly higher than that of TXT [43]. Petersen et al. determined that surfactants can improve nanocarrier adsorption by interaction with cell membranes, and can facilitate endocytotic pathways. Table 4 shows the IC_50_s of the prepared formulations and TXT. A concentration of 0.044 µM of Formulation I acted similarly to a concentration of 0.093 µM of TXT. Results demonstrate that in order to lower the IC_50_, the efficacy of formulations I and III is higher than that of TXT in a similar period (after 72 h). It seems that micelle degradation by lipase enzyme releasing from C26 cells leads to complete DTX release near the cells. In addition, surfactant can inhibit P-glycoprotein and multidrug resistance (MDR) mechanisms, and accumulate drug in the cancer cells [44]. Furthermore, the IC_50_ can be better than that of commercial products due to direct drug delivery to the cytosol.

Normally, three mechanisms of swelling/erosion, diffusion, and degradation have been presented for drug release (uptake) from carriers [45]. One or all of these mechanisms may occur in nanosystems, and water uptake rate in release behavior is determined by the hydrophilicity of surfactants [10]. Water uptake leads to nanomicelle swelling, and loaded drug in the core of carrier is diffused through the pores. Cellular uptake studies revealed that DTX uptake from Formulation I by the cell was significantly higher than for TXT. One of the most important challenges in oral administration of drugs is the limitations of the gastrointestinal tract including media pH, enzymatic dissolution, and poor epithelium permeability. The gastrointestinal tract pH can vary from 1 in the stomach to 8 in the intestine. This pH variation leads to oxidation, deamination, or hydrolysis of drug proteins, and decreases their activity. If a drug overcomes these issues, it can reach epithelial cells for absorption. A transcellular pathway mechanism is used for DTX intake. A vesical-like substance is created by the folding and closing of plasma membranes of the absorbing cells which can transfer DTX nanomicelles into the cell [40].

## 4. Materials and Methods

### 4.1. Materials

DTX was purchased from Knowshine Pharmachemical Inc. (Shanghai, China), Tween 20 and Tween 80 were from Merck (Darmstadt, Germany), and MCT oil was from the SHS Company (Belfast, UK). Water was distilled twice, and sterilized by passing through 2 µm filters. Taxotere^®^ was purchased from Sanofi Aventis (Paris, France) in vial form. Methanol, ethanol, acetonitrile, and chloroform were HPLC grade. Enteric liquid phase has prepared using sodium chloride from Merck, sodium dodecyl sulfate from Sigma Aldrich Company (Darmstadt, Germany), HCl from Merck, dipotassium hydrogen phosphate from Sigma Aldrich Company, potassium chloride (Merck), sodium taurocholate lecithin from Sigma Aldrich and HEPES.

### 4.2. DTX-Loaded Nanomicelles Preparation

Tween 20 and Tween 80 (both 55% wt.), MCT oil 20% wt., and distilled water 24% wt. were used to prepare two DTX-loaded nanomicelles. All materials were weighed in 15 mL Falcon tubes, and were heated up to 50 °C in a hot water bath. Agitation was performed until complete dissolution with sequence of 4 min. intervals by a vortex device (Reax, Heidolph, Schwabach, Germany). After 30 min, the solution was cooled down to 37 °C and 1% of DTX was added and the solution was stored in 37 °C for 40 min. Continuous vortexing was used to mix the solution. In case a homogenous solution was not achieved after 30 min of agitation, it was ultra-sonicated in a proper cycle for 1 to 2 min (Elma S60H, Elma Schmidbauer GmbH, Singen, Germany).

Nanomicelles with the two surfactants Tween 80 and 20 without DTX were prepared in the same way control the empty nanomicelle was prepared. Figure 7 shows the prepared nanomicelle solution contacting DTX, which is a completely clear solution.

### 4.3. Nanomicelles Characterization

Transmission electron microscopy (EM10C TEM, Ziess, Oberkochen, Germany) was used to determine the structure and morphology of nanomicelles. In order to prepare the samples, a few droplets of the prepared formulation were placed on formvar-carbon coated cupper grid mesh 300, and air dried. Then the nanomicelles were viewed and photographed with TEM.

The mean diameter of nanomicelles and polydispersity index (PDI) were determined using a Dynamic Light Scattering Instrument (Nano-ZS; Malvern, Malvern, UK) in triplicate. The zeta potential of liposomes was determined on the same equipment by using the zeta potential mode averaging 20 measurements. Particle sizes were reported as the means ± standard deviation and polydispersity index (*n* = 3). Zeta potentials were reported as the means ± zeta deviation (*n* = 3).

### 4.4. Determination of Encapsulation Efficiency of DTX

The amount of drug DTX loaded into the formulation was determined by HPLC (Waters, Milford, MA, USA) using a reverse-phase stainless steel C18 column (4.6 × 250 mm^2^) to the pharmacopeial method (USP 39). The mobile phase was acetonitrile and water (55:45 *v*/*v*), with 1 mL/min flow at room temperature. To draw calibration curves, DTX (0.001 to 0.1 mg/mL) was dissolved in the methanol and acetonitrile (1:1 *v*/*v*), and 20 µL of the solution was injected into the HPLC instrument. To determine the concentration of DTX, nanomicelles were dissolved in the methanol and then injected into the HPLC. In order to determine the encapsulation efficacy, an aliquot of prepared formulation was poured into a plastic conical tube (Amicon, Billerica, MA, USA) with 50,000 Mw cut-off and centrifuged in 1500 rpm for 4 h to separate the un-encapsulated DTX. Then the supernatant was analyzed by HPLC, and the DTX concentration was measured. Encapsulation efficacy (EE) of DTX concentration in system has been determined through Equation (1):

(1) $$EE\%=\frac{Actual\;concentration\;of\;DTX\;in\;the\;formulation}{Initial\;concentration\;of\;DTX}\times 100$$

### 4.5. Stability Studies

Stability studies were carried out in three different conditions by particle size measurement and zeta potential analysis; condition A: for the first 24 h after producing sample at room temperature with 4 h intervals, condition B: under real storage condition of 2–8 °C at 1, 2, 3, 4 weeks, and 3, 6, 9, and 12 months after preparation, and condition C: in simulated gastric fluid and simulated intestinal fluid at 2, 4, 6, 8, and 12 h.

Simulated gastric fluid (SGF, pH 1.6) and simulated intestinal fluid (SIF, pH 6.5) were used to evaluate the stability of the formulations for oral administration [46]. SGF was prepared from 0.2% sodium chloride (NaCl), 0.25% sodium dodecyl sulfate (SDS), and 0.7% hydrochloric acid (HCl) in water, and pH was adjusted to 1.6. SIF was prepared from 0.3% dipotassium hydrogen phosphate (K_2_HPO_4_) with 0.77% potassium chloride, 3 mM sodium taurocholate, and 0.75% mM lecithin. Prepared formulations were mixed with appropriate volume of SGF or SIF and the stability studies was conducted at 37 °C [10]. In addition, EE evaluated for prepared formulations by HPLC analysis in condition (A) at room temperature and after 24h and in condition (B) 2–8 °C after 12 months.

### 4.6. In Vitro Cells Toxicity Studies

Cultured C-26 cells were used to analyze the formulation efficacy by the MTT method and to compare it with TXT. Cancer cells (5000 cells) were seeded in a 96 well plate and stored to form surface binding for 24 h at 37 °C under 5% CO_2_. Then the specified amount of formulation was added to each sample three times. As a reference 0.1 µg/mL concentration of TXT was used. Added medium in each well was 200 µL, and after 48 h of incubation, the previous medium was replaced with 100 µL of RPMI 1640 without serum, plus 0.1% of the well volume of MTT solution (5 mg/mL buffered saline solution of PBS-phosphate), and kept in the incubator for 2–4 h at 37 °C. Then cultured medium was removed from each well and 100 µL DMSO was added. Absorbance of solution was measured by an ELISA reader with 570 nm wavelength. Blank cells were considered as the control with 100% viability, and cells without MTT were considered as the blank to calibrate the spectrophotometer with zero absorption. Viability percent was calculated by Equation (2):

(2) $$Viability\;\%=\frac{Absorbance\;of\;each\;well}{Mean\;absorbance\;of\;wells\;without\;changes}\times 100$$

The half maximal inhibitory concentration (IC_50_) was calculated by plotting viability (y-axis) vs. log C (µM, x-axis).

### 4.7. Cellular Uptake

In order to calculate cellular uptake, C-26 cell lines were incubated in a 24-well plate for 12 h. Then, the medium was removed and 1 mL of PBS solution was added to each well, and dead or floating cells were removed. Afterwards, 1 mL of formulation and 100 µg/mL of TXT were added to each well. Culturing was continued for 4, 12, and 24 h at 37 °C under 5% of CO_2_. Finally, each well was rinsed with PBS three times to remove all remaining formulation and TXT. Two hundred µL of trypsin was added to each well and kept for 15 min, then all cells were detached from the plate, 80 µL of methanol/acetonitrile was added to each well and it was agitated for 3 min. After that, each plate was stored for 48 h in 4 °C to ensure complete degradation and extraction. Contents of each well was gathered into a microtube, agitated for 2 min by a vortex device, and centrifuged for 20 min in 1000 rpm at 4 °C. Finally, 20 µL of supernatant was assayed for DTX by the HPLC method.

## 5. Conclusions

In this study, DTX-loaded nanomicelles were successfully formulated. Nanomicelles increased the water solubility of DTX more than 1500 times (10 mg/mL in nanomicelles compared to 6 µg/mL in water). The prepared nanomicelles with particle size of approximately 14 nm and encapsulation efficiency of 99%, were stable in gastric fluid and intestinal fluid for at least 6 h and decreased the IC_50_ significantly after 72 h exposure compared to Taxotere^®^. Since the nanomicelles increased the water solubility of DTX, they can bypass the unstirred water layer barrier of GIT, hence increasing the oral absorption of DTX. Therefore, the results of this study revealed that the proposed new nanomicelle formulation of DTX could be used for the oral delivery of DTX and merits further investigation.

## Figures and Tables

**Table 1:** Formulation Content.

Formulation	Content
I	DTX, Tween 80, MCT oil, water
II	Tween 80, MCT oil, water
III	DTX, Tween 20, MCT oil, water
IV	Tween 20, MCT oil, water

**Table 2:** Particle size (Z-average (nm)), PDI, and zeta potential (mV) of formulations (mean ± SD, *n* = 3).

Formulation	Z-Average (nm)	PDI	Zeta Potential (mV)
I	14.03 ± 1.23	0.132 ± 0.02	−9.45
II	9.89 ± 2.68	0.125 ± 0.07	−5.67
III	132.55 ± 12.88	0.256 ± 0.02	−6.09
IV	126.2 ± 19.39	0.224 ± 0.06	−2.97

**Table 3:** Encapsulation efficacy (EE %) of the DTX in the nanomicelles for long term and short term stability studies.

Condition	Time	EE %
A (at room temperature)	After 24 h	99 ± 0.05
B (at 2–8 °C)	After 12 months	98.9 ± 0.1

**Table 4:** IC_50_ (µM) for formulations and TXT, on C-26 cell line after 72 h.

Formulation	IC_50_ (µM)
I	0.044853
III	0.061314
TXT	0.093071
